# Molecular epidemiology of enteroviruses from Guatemalan wastewater isolated from human lung fibroblasts

**DOI:** 10.1371/journal.pone.0305108

**Published:** 2024-07-03

**Authors:** Leanna Sayyad, Chelsea Harrington, Christina J. Castro, Hanen Belgasmi-Allen, Stacey Jeffries Miles, Jamaica Hill, María Linda Mendoza Prillwitz, Lorena Gobern, Ericka Gaitán, Andrea Paola Delgado, Leticia Castillo Signor, Marc Rondy, Gloria Rey-Benito, Nancy Gerloff

**Affiliations:** 1 Contracting Agency to the Division of Viral Diseases, Cherokee Nation Assurance, Tulsa, Oklahoma, United States of America; 2 Division of Viral Diseases, Polio and Picornavirus Branch, U.S. Centers for Disease Control and Prevention, Atlanta, Georgia, United States of America; 3 Contracting Agency to the Division of Viral Diseases, IHRC Inc., Atlanta, Georgia, United States of America; 4 Ministerio de Salud Pública y Asistencia Social Guatemala, Guatemala City, Guatemala; 5 Pan-American Health Organization/World Health Organization, Guatemala Country Office, Guatemala City, Guatemala; 6 Pan-American Health Organization, World Health Organization, Washington, DC, United States of America; Khoo Teck Puat Hospital, SINGAPORE

## Abstract

The Global Specialized Polio Laboratory at CDC supports the Global Poliovirus Laboratory Network with environmental surveillance (ES) to detect the presence of vaccine strain polioviruses, vaccine-derived polioviruses, and wild polioviruses in high-risk countries. Environmental sampling provides valuable supplementary information, particularly in areas with gaps in surveillance of acute flaccid paralysis (AFP) mainly in children less than 15 years. In collaboration with Guatemala’s National Health Laboratory (Laboratorio Nacional de Salud Guatemala), monthly sewage collections allowed screening enterovirus (EV) presence without incurring additional costs for sample collection, transport, or concentration. Murine recombinant fibroblast L-cells (L20B) and human rhabdomyosarcoma (RD) cells are used for the isolation of polioviruses following a standard detection algorithm. Though non-polio-Enteroviruses (NPEV) can be isolated, the algorithm is optimized for the detection of polioviruses. To explore if other EV’s are present in sewage not found through standard methods, five additional cell lines were piloted in a small-scale experiment, and next-generation sequencing (NGS) was used for the identification of any EV types. Human lung fibroblast cells (HLF) were selected based on their ability to isolate EV-A genus. Sewage concentrates collected between 2020–2021 were isolated in HLF cells and any cytopathic effect positive isolates used for NGS. A large variety of EVs, including echoviruses 1, 3, 6, 7, 11, 13, 18, 19, 25, 29; coxsackievirus A13, B2, and B5, EV-C99, EVB, and polioviruses (Sabin 1 and 3) were identified through genomic typing in NGS. When the EV genotypes were compared by phylogenetic analysis, it showed many EV’s were genomically like viruses previously isolated from ES collected in Haiti. Enterovirus occurrence did not follow a seasonality, but more diverse EV types were found in ES collection sites with lower populations. Using the additional cell line in the existing poliovirus ES algorithm may add value by providing data about EV circulation, without additional sample collection or processing. Next-generation sequencing closed gaps in knowledge providing molecular epidemiological information on multiple EV types and full genome sequences of EVs present in wastewater in Guatemala.

## Introduction

Enteroviruses (EV) account for an estimated 10–15 million symptomatic infections in the United States each year [1]. EVs form one genus of the *Picornaviridae*, a family of small, non-enveloped, positive-strand RNA viruses that are globally widespread [2]. EVs are composed by nearly 100 serotypes of coxsackievirus, echovirus, and poliovirus, and are transmitted by (1) fecal-oral, (2) respiratory droplet, and (3) water, food, and fomites [3]. EVs in the environment are mainly found in water sources, including sewage systems, causing diseases that can present with neurological complications [4]. EVs that are shed into water can be transmitted to people in the area that come in contact with contaminated water [5].

EVs are highly prevalent and are associated with a wide range of diseases, particularly affecting infants, and young children. EVs can cause a range of diseases, from asymptomatic infections to mild respiratory illness, aseptic meningitis in neonates, acute flaccid paralysis, and death [5]. Recently, Europe and the US have seen a rise of cases of myocarditis in neonates caused by EVs, including coxsackievirus B3 and B4 [6]. EV-A71 is responsible for a majority of severe cases and some fatalities in several regions of the world that led to the development of a vaccine in China [7]. Wastewater surveillance can uncover enteroviruses circulating in a community, in the absence of clinical data for EV infections, adding public health value [8]. Furthermore, the identification of genetic diversity, such as mutation and recombination, in EVs can offer pertinent insights into their evolution and virulence [8].

ES is essential for monitoring EVs, including polioviruses (PVs), although this study extends beyond PVs [9]. Though Guatemala’s absence of wild-type polio cases since 1990, the risk of PV outbreaks persists due to vaccination coverage rates below 95% [10]. Collaborative efforts between the Pan American Health Organization (PAHO) and the Guatemala Ministry of Health (MoH) have established robust surveillance systems, particularly in two major cities (San Juan Sacatepéquez and Villa Nueva), utilizing six ES collection sites. Notably, these same sites serve as collection sites for this study, aimed at testing and isolating EVs to enhance the surveillance system. Wastewater-based PV surveillance in Guatemala detected vaccine-derived polioviruses (VDPVs) in 2019, prompting an enhanced response [11]. Regular vaccination and targeted immunization campaigns maintain high vaccination rates, while a robust ES program effectively monitors enteroviruses in the community. Enhancing the monitoring of a variety of EVs through this PV surveillance can yield cost-sharing benefits and improve the overall effectiveness of Enterovirus Surveillance [12].

EVs can be cultured in various mammalian cell lines, with different serotypes exhibiting varying growth abilities [13]. To achieve comprehensive EV detection, it is crucial to include cell lines capable of capturing additional EVs that may not grow in the PV-selective L20B cells or in EV-permissive RD cells. These cell lines are considered the gold standard in PV wastewater and AFP surveillance testing and were chosen for their high susceptibility to polioviruses. However, L20B and RD cells may not encompass the complete range of enteroviruses found in sewage samples, as EV represents a diverse group with many serotypes extending beyond polioviruses. In a ES study in Haiti, it was shown that the use of these two cell lines (L20B and RD) was effective in isolating multiple echoviruses, and it emphasized the diverse nature of EVs underscoring the potential for a comprehensive pan-enterovirus surveillance [14]. Next-generation based amplicon sequencing (NGS) has been used to successfully detect viruses in sewage [15], including SARS-CoV-2 [16], EVs [17,18], norovirus [19,20], adenovirus [21], and human sapovirus [22]. NGS has the advantage of high sensitivity and high throughput for detecting viruses in mixed samples and can detect less prevalent genotypes that are undetectable by Sanger sequencing [22].

Building upon available data, we introduced an additional cell line, human lung fibroblasts (HLF), combined with NGS and molecular typing to enhance EV detection and supplement existing poliovirus surveillance on a selection of ES samples. The objective of this study was to leverage the existing wastewater testing in determining if (i) adding another cell line can capture additional EV’s not present in RD isolates, (ii) if EV typing and the molecular epidemiology from ES isolates shows presence of human pathogenic EV’s and (iii) to inform if EV circulation varies in Guatemala throughout the year without collecting additional samples. NGS results showed a diversity of enteroviruses captured by wastewater surveillance by adding an HLF cell line to the poliovirus ES testing algorithm.

## Materials and methods

### Water sample collection

For PV surveillance, one liter of influent wastewater was collected from six previously established sites in Guatemala and with five sites defined as open canals [23]. The six sites were located in Guatemala’s urban municipalities of San Juan Sacatepéquez: Cuidad Quetzal (CQU), Cerro Candelaria Bodega Municipal (CBM), Aldea Cruz Blanca (ACB), and Villa Nueva: Rio Platanitos (PLA), Colina de Villa Nueva (CVP), and Rio San Lucas (RMG). Each site was selected with a catchment area of around 100,000 individuals. The grab sampling method with swing sampler collection was selected since the sites were either open waters or streams [23]. The water samples were collected monthly starting November 2018 and ongoing as recommended by GPLN/WHO [9]. The wastewater samples were stored in collection bottles at 2–8°C immediately after collection for transportation to the National Health laboratory and stored at -20 °C until shipment on dry ice to the processing lab (CDC, Atlanta). All specimens were stored at -80°C until processing. This study utilized a subset of samples collected between April 2020 and September 2021.

### Preliminary cell line selection and parallel testing

To conduct a pilot assessment, a set of five cell lines were chosen based on their sensitivity to enteroviruses, informed by relevant literature research, and considering their availability at the CDC. The cell lines included African green monkey kidney epithelial (Vero, ATCC# CCL-81, CRL-1586), human epithelia (HEP-2, CCL-23), African green monkey kidney (MA-104, CRL-2378.1), adenocarcinomic human alveolar basal epithelial (A-549, ATCC# CCL-185), and human lung fibroblast (HLF, CCL-199) cells. Cells were chosen for the isolation and characterization of NPEVs and compared to the rhabdomyosarcoma (RD, ATCC # CCL-136) cell line. L20B cells were not selected for comparison because they are highly susceptible to PV infection. All cell lines were seeded in 75-cm^2^ flasks containing Eagle’s minimum essential media (MEM) supplemented with 10% (growth medium) heat inactivated Hyclone fetal bovine serum (FBS; Atlanta Biologicals, Atlanta, GA) and 100 IU mL^-1^ penicillin, 10 μg mL^-1^ streptomycin (Sigma Aldrich, St. Louis, MO). A concentration of 2.0 x 10^5^ cells/ml were seeded and grown for three days at 36°C until 75% to 90% confluency [24]. The six selected sites served as monitoring locations due to Guatemala’s existing surveillance system for poliovirus, and concentrated ES samples were processed from each site. Each cell line was inoculated and examined for cytopathic effects (CPE). Next-generation sequencing (NGS) was performed using the Illumina MiSeq platform from CPE-positive isolates. The results of NGS were compared to the sequencing outcomes of the RD cell line, resulting in the preference of the HLF cell line using this preliminary study.

### Sample concentration and inoculation

Environmental sewage samples were concentrated using the CAFÉ filter-elution procedure [23]. Using a vacuum pump, the samples passed through two 47 mm filters made of mixed cellulose ester (Advantec, Chiyoda City, Tokyo, Japan). The first phase filtered the samples using 5 μm filters and the second phase using 0.45 μm filters that were placed in beef extract (3% w/v). Supernatants were used as concentrates.

### Virus isolation and rRT-PCR verification

All viruses were isolated according to the recommended WHO PV isolation protocol, using RD cells [24]. On the day of inoculation, media in flasks were changed from 10% FBS growth to 2% FBS maintenance media with antibiotics (100 IU mL-1 penicillin, 100 μg mL-1 streptomycin, and 50 μg mL-1). Concentrates were inoculated into each cell line, placed into an incubator at 36°C and 5% CO_2_ except for HLF which was incubated at 33°C and 5% CO_2_, and observed for CPE over the course of five days using a Axio Zeiss microscope (with camera). HLF cells resemble smooth muscle cells but have prominent features of long branching processes [25]. A negative flask, a flask with little to no CPE, would give the muscle-like resemblance with no visible effect, while a positive flask, a flask with CPE of 3 or more, would appear with rounded refractile cells detached from the flask’s surface showing visible damage in cells caused by viral infection (S1 Fig). All CPE-positive samples were screened for the presence of NPEVs and polioviruses using real-time RT-PCR using the intratypic differentiation (ITD) kit for poliovirus [26,27].

### Next-generation sequencing

Total nucleic acid (TNA) was extracted through Kingfisher automatic extraction system using the MagMax pathogen RNA/DNA kit (ThermoFisher Scientific, Waltham, MA) according to the manufacturer’s instructions and followed with DNase treatment (ThermoFisher Scientific, Waltham, MA). Random reverse transcription was performed using a 28-base oligonucleotide whose 3’ end consisted of eight Ns (all four nucleotides at each of the eight 3’ positions) and whose 5’-end 20 bases consisted of an arbitrarily designed sequence (primer N1) [28]. A second-strand cDNA synthesis was performed using Klenow fragment DNA polymerase extension (New England BioLabs, Ipswich, MA). PCR amplification was followed using AmpliTaq Gold DNA polymerase and a 20-base primer (ThermoFisher Scientific, Waltham, MA). The randomly amplified nucleic acid was then subjected to the NexteraXT library preparation protocol (Illumina, San Diego, CA) according to the manufacturer’s instructions and sequenced using Illumina’s Miseq platform [28]. The amplicons were visualized by electrophoresis on an agarose gel or on a TapeStation (Agilent Technologies, Santa Clara, CA) for selecting appropriate quantities of DNA for downstream applications [18].

### Genome sequence and phylogenetic analysis of enteroviruses

A custom in-house bioinformatics pipeline, VPipe, was used to process raw reads and *de novo* assemble each isolate [29]. There were multiple preprocessing steps within the pipeline: 1) raw sequencing reads were filtered to remove host DNA sequences using Bowtie2 v2.3.3.1; 2) reads were trimmed to remove primers and adapters; a Phred quality score filter of q20; an error rate cutoff of 0.15 and a minimum read length of 50 nucleotides was applied using Cutadapt v2.3, and 3) duplicate reads were removed using the Dedup.py script in Python [30–32]. The remaining reads were then *de novo* assembled to produce sequence contigs using SPAdes v3.15.0 [33]. Consensus genome sequences were verified through read mapping and annotations using Geneious vR11 [34], alignments using MAFFT v7.450 [35] and NCBI’s BLAST [36] results. Full coverage of the typing region (VP1) with a minimum average coverage of 25X was used for typing. A total of 21 EVs near complete whole genomes were aligned using MAFFT (default parameters), followed by neighbor joining phylogenetic analysis using the PAUP* v4.0 [37] plugin in Geneious. The PAUP* parameters were as follows: tree building method = neighbor joining, genetic distance model = GTR, rates variation = gamma distribution, outgroup = none, and root method = midpoint. Genome sequences (5 partial genomes and 16 complete genomes) were deposited in NCBI’s GenBank sequence database, accession numbers OL955504-512 and ON38146-157 (Table 2). All statistical and visual results were compiled and managed in R [38] and data visualizations were made using the ggplot2 [39] package in R.

### Ethical considerations

CDC’s internal program for Human Subjects Research Determination deemed that this study is categorized as public health non-research and that human subject regulations did not apply.

## Results

### Preliminary selection of cell lines

**Fig 1:**
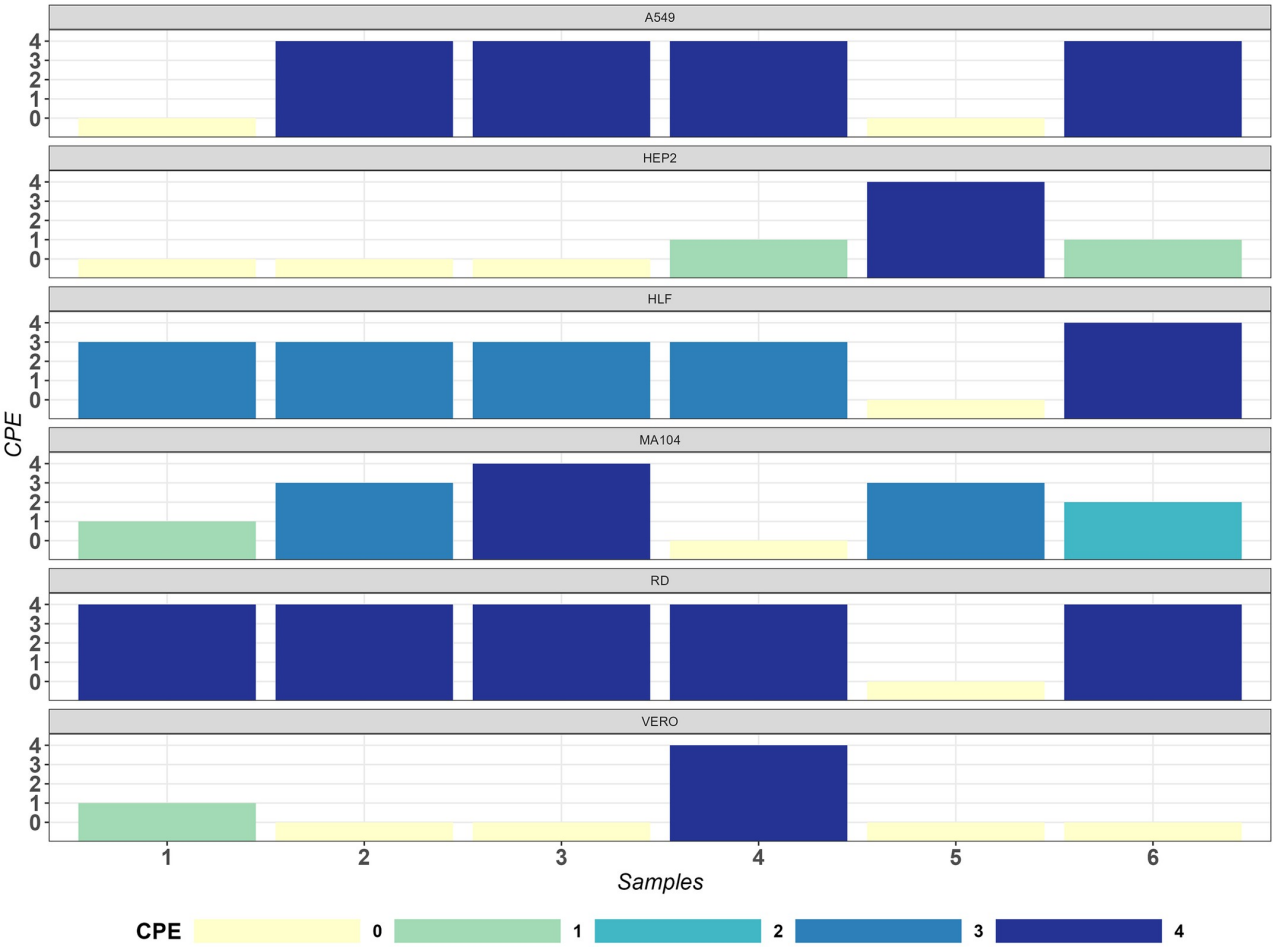
CPE (Cytopathic effect) results between the six cell lines. CPE graded 0 through 4 for A549, HEP2, HLF, MA104, RD and Vero cell lines for samples 1 through 6.

**Fig 2:**
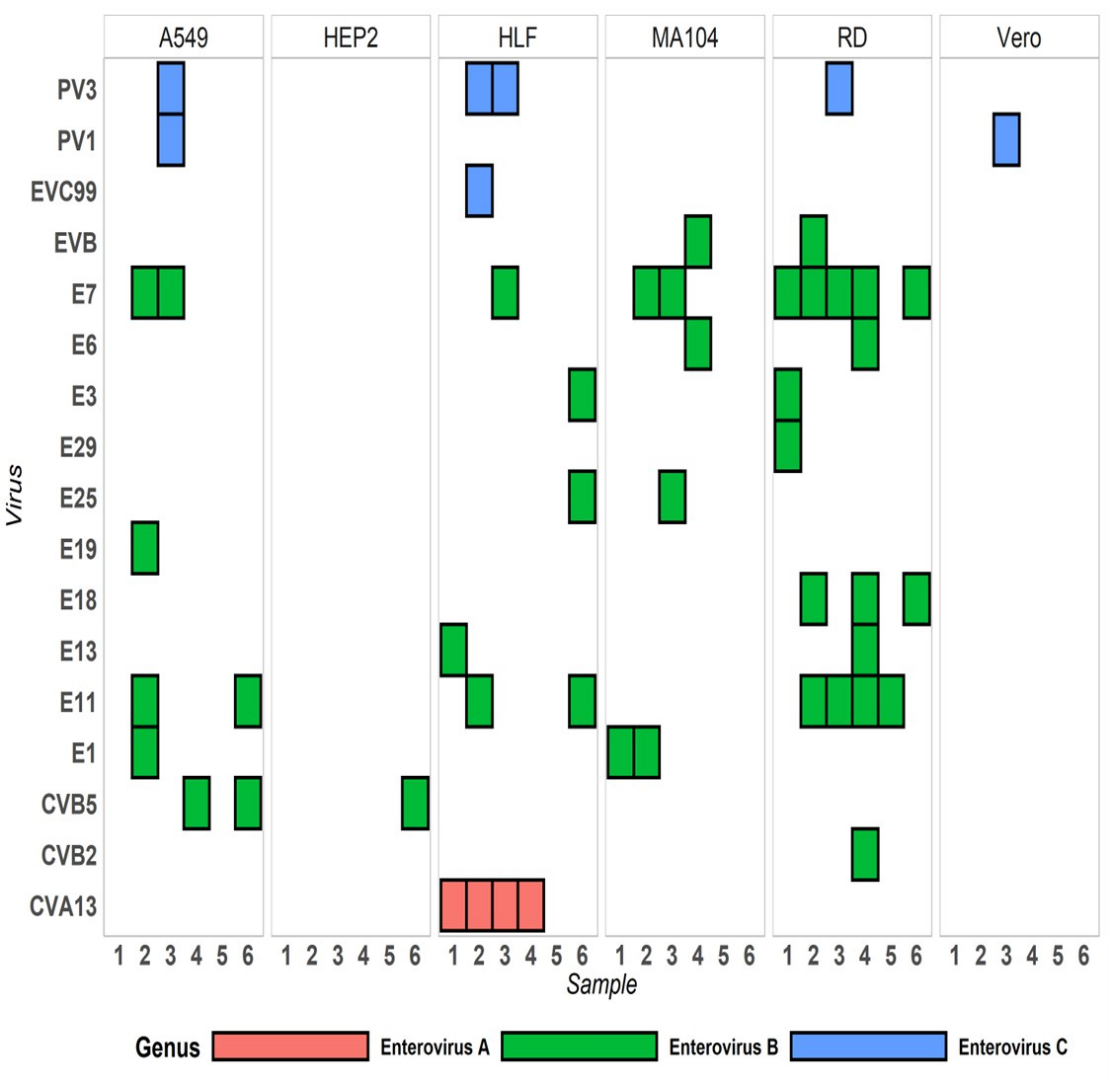
Enterovirus typing from preliminary NGS of 6 cell lines. The A549, HEP2, HLF, MA104, RD and Vero cell lines identified polioviruses (PV3, PV1), echoviruses (E1, E3, etc.), Coxsackieviruses (CV) B5, B2 and CVA13. Color coding shows genus: red—Enterovirus A, green—Enterovirus B, blue—Enterovirus C.

Microscopic observation confirmed alterations in cell monolayers to determine cytopathic effects (CPE) and capture images (S1 Fig). During the pilot assessment, the HLF cell line exhibited consistent CPE outcomes compared to RD cells, with positive results observed in all six samples, except for sample 5 (Fig 1). The A549 cells exhibited CPE positivity in samples 2, 3, 4, and 6, while MA104 showed positive results for samples 2, 3, 5, and 6. On the other hand, the HEP2 and Vero cell lines demonstrated only one instance of CPE positivity, observed in samples 5 and 4, respectively (Fig 1). EVs were categorized into four groups to determine if a cell line would be more susceptible than the RD cell line. HEP2 cells only selected for Coxsackievirus B (CVB), while Vero cells only selected for PV. MA-104 cells selected for Echoviruses (E) and enterovirus B (EVB). HLF, A549 and RD cells all selected mixtures of PV, E, and CVB (Fig 2). HLF cell line were also the only cell line to select for Coxsackievirus A (CVA). RD, the gold standard for enteroviruses, which serve as a surrogate for poliovirus, requires any cell line to match its sensitivity or enhance the value of the standard PV cell culture procedures. Notably, the L20B cell line was not selected as a comparator due to its focus on polioviruses. Microscopic observation confirmed alterations in HLF cell monolayers to determine cytopathic effects (CPE) and capture images (S1 Fig).

### Preliminary genome and phylogenetic analysis of Enteroviruses

**Fig 3:**
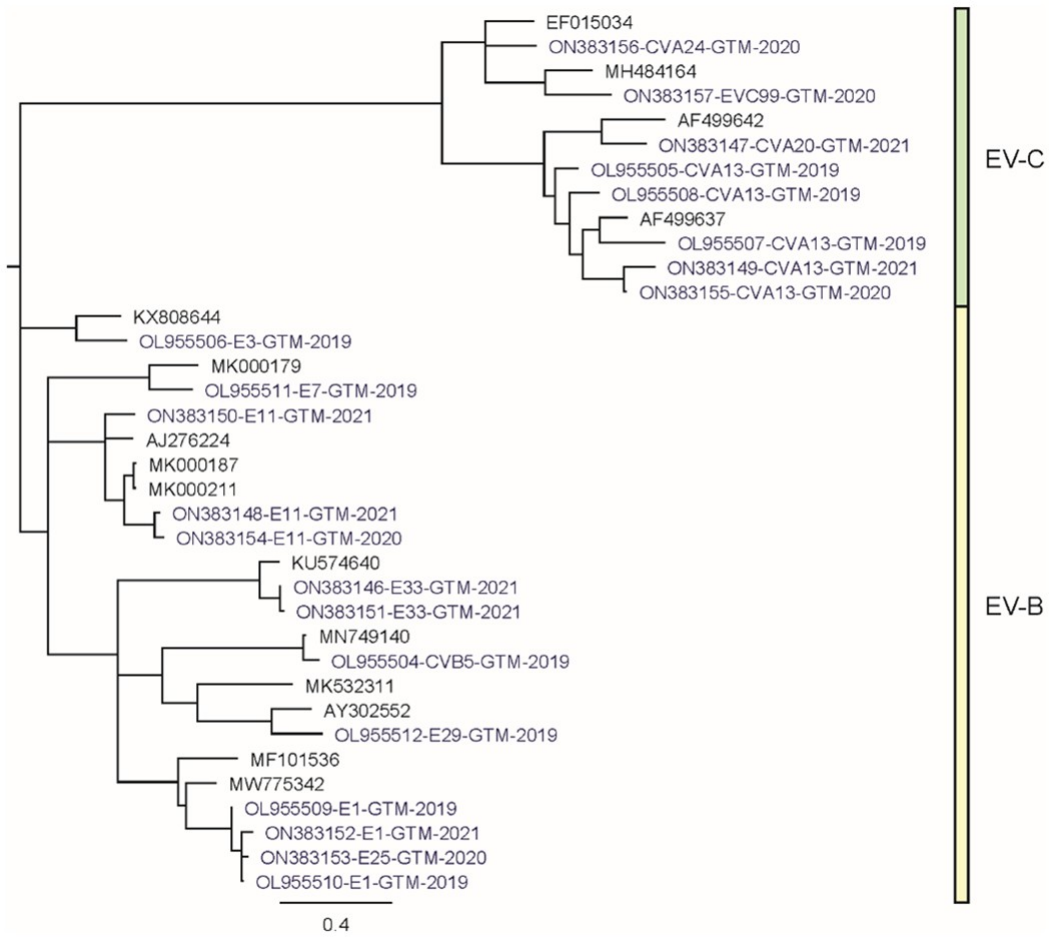
Phylogenetic relationship of the Viral Protein 1 (VP1) typing region of 21 enterovirus sequences from Guatemala and 15 representative genomes of each type using a PAUP* neighbor joining tree building algorithm. Tree created using MAFFT global alignment (default parameters) and PAUP* phylogenetic analysis software. The PAUP* parameters were as follows: tree building method = neighbor joining, genetic distance model = GTR, rates variation = gamma distribution, outgroup = none, and root method = midpoint.

Sequence comparison of EV genomes found in the preliminary study revealed complex mixtures among the six ES samples (Table 1). The HLF cell line detected three different EV species (A, B, and C) (Fig 1). The viruses detected within the HLF cell line were echovirus E3, 7, 11, 13, and 25, EV-C99, poliovirus type 3 (PV3), and CVA-13. A549 cell isolates contained genomic RNA that resulted in the detection of echoviruses 1, 7, 11, and 19, CVB-5, PV1, and PV3. The MA104 cell line detected E1, E6, E7, and E25. The HEP2 and Vero cells lines detected the least number of viruses, enterovirus species C; CVB-5 and PV1, respectively (Fig 1, Table 1). The viral protein 1 (VP1) typing region of each sequence from Table 2 was used for the phylogenetic tree, with poliovirus genomes excluded (Fig 3). The CVAs and EVCs clustered together in one clade as they are all species enterovirus C, while all the echoviruses clustered together in a separate clade as they belong to species enterovirus B. All sequences were confirmed by VP1 cluster analysis as described in 1.6 above. The 5 additional EV genomes, presented in Table 2, constitute the new genome submissions included in this publication [40].

**Table 1:** Identification of EV serotypes by genome comparison in preliminary study.

Sample	A549	HEP2	HLF	MA104	RD	Vero
1			E13, CVA-13	E1	E3, E7, E29	
2	E1, E7, E11, E19		PV3, CVA-13, EV-C99, E11	E1, E7	E7, E11, E18, EVB	
3	E7, PV1, PV3		CVA-13, E7, PV3	E7, E25	E7, E11, PV3	PV1
4	CVB-5		CVA-13	EVB, E6	E6, E7, E11, E13, E18, CVB-2	
5					E11	
6	E11, CVB-5	CVB-5	E3, E11, E25		E7, E18	

Six samples from Guatemala sites with complex mixtures revealed among the six cell lines. Abbreviations: CV-Coxsackievirus, E-Echovirus, EV-Enterovirus, PV-poliovirus.

**Table 2:** Table of 21 EV serotypes sequenced using NGS and submitted to NCBI’s GenBank database.

GENBANK ACCESSION NO.	SEROTYPE	SPECIES	CELL LINE	COLLECTION DATE	LENGTH (NT)	TOTAL NO. OF MAPPED READS	GC CONTENT
**OL955504**	Coxsackievirus B5	*Enterovirus B*	A549	11/22/2019	7302	9553	47.80%
**OL955505** *	Coxsackievirus A13	*Enterovirus C*	HLF	11/20/2019	7015	2139	44.70%
**OL955506**	Echovirus E3	*Enterovirus B*	HLF	11/20/2019	7342	15035	47.80%
**OL955507**	Coxsackievirus A13	*Enterovirus C*	HLF	11/22/2019	7395	23120	45.30%
**OL955508** *	Coxsackievirus A13	*Enterovirus C*	HLF	11/20/2019	7394	5890	45.10%
**OL955509**	Echovirus E1	*Enterovirus B*	MA104	11/20/2019	7132	6015	47.90%
**OL955510** *	Echovirus E1	*Enterovirus B*	MA104	11/20/2019	7143	3891	47.30%
**OL955511**	Echovirus E7	*Enterovirus B*	MA104	11/20/2019	7270	14881	47.50%
**OL955512**	Echovirus E29	*Enterovirus B*	RD	11/20/2019	7314	4717	47.80%
**ON383146**	Echovirus E33	*Enterovirus B*	HLF	09/01/2021	7240	33169	47.90%
**ON383147**	Coxsackievirus A20	*Enterovirus C*	HLF	05/18/2021	7185	4154	45.80%
**ON383148** *	Echovirus E11	*Enterovirus B*	HLF	05/18/2021	7140	6647	47.50%
**ON383149**	Coxsackievirus A13	*Enterovirus C*	HLF	05/18/2021	7318	57563	45.10%
**ON383150**	Echovirus E11	*Enterovirus B*	HLF	04/16/2021	7182	7861	47.70%
**ON383151** *	Echovirus E33	*Enterovirus B*	HLF	04/16/2021	7080	1900	48.10%
**ON383152**	Echovirus E1	*Enterovirus B*	HLF	01/25/2021	7211	3399	47.30%
**ON383153**	Echovirus E25	*Enterovirus B*	HLF	09/16/2020	7259	9325	47.60%
**ON383154**	Echovirus E11	*Enterovirus B*	HLF	07/13/2020	7312	35931	47.50%
**ON383155**	Coxsackievirus A13	*Enterovirus C*	HLF	06/10/2020	7355	17008	44.40%
**ON383156**	Coxsackievirus A24	*Enterovirus C*	HLF	06/10/2020	7365	13212	44.70%
**ON383157**	Enterovirus C99	*Enterovirus C*	HLF	05/15/2020	7302	8644	44.90%

Twenty-one enterovirus samples sequenced using next-generation sequencing Illumina MiSeq submitted to NCBI’s GenBank database and used in the phylogenetic analysis in Fig 3.

*These accession numbers denote the 5 genomes that were submitted as part of this publication. The 16 other genomes were submitted previously [41]. Accession numbers prefixed with "OL" correspond to the preliminary study, whereas those beginning with "ON" denote results from testing exclusively with the HLF cell line.

### Prospective sample testing and genomic EV sequences in HLF cell line

One hundred and four ES samples collected from April 2020 to September 2021 were inoculated into the HLF cells and compared to the previously inoculated RD cell isolation results. Sixty-three samples showed CPE-positive results (CPE 3–4) in both the HLF and RD cell lines. One sample was positive only in the HLF cell line, and 40 samples were positive only in RD cells. The 63 CPE-positive samples in the HLF cell line were sequenced using NGS. Of the 63 samples tested, 58 contained at least one EV (Table 2).

### Seasonality and geographic distribution of EVs

**Fig 4:**
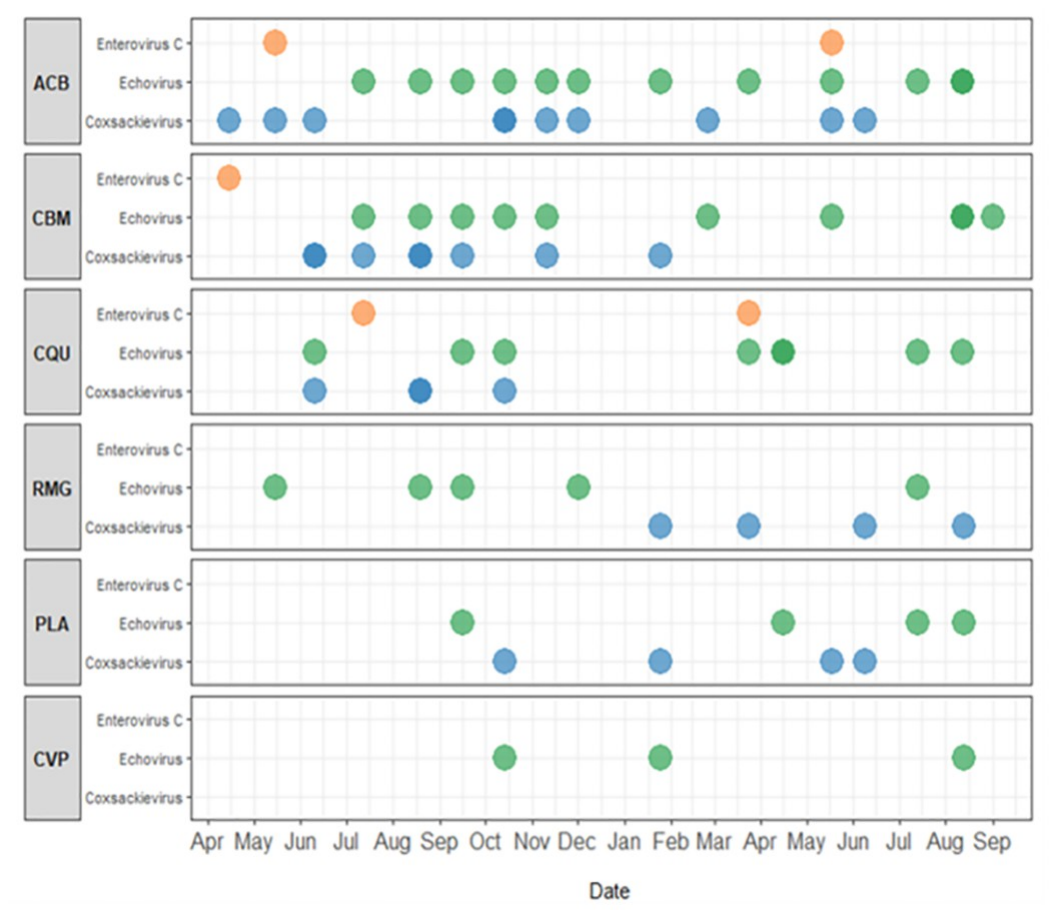
Detection patterns of enterovirus C (poliovirus, orange circles), echovirus (green), Coxsackievirus (blue) in Guatemala by collection site (ACB, CBM, CQU-San Juan Sacatepéquez. RMG, PLA, CVP-Villa Nueva) and by month of collection (April 2020 -September 2021), 63 samples were tested using the HLF cell line and 58 samples contained at least one enterovirus through sequence comparison.

The six preliminary samples plus the 63 HLF positive samples collected in a span of 18 months showed that most echoviruses and Coxsackieviruses circulated annually (between years 2020–2021). The identification of Sabin-like poliovirus (oral polio vaccine virus) are likely from immunization campaigns in Guatemala. Enterovirus C99 was detected at three sites (ACB, CBM, RMG) during the wet season, typically between May-October. Echoviruses, E11, E13; E25, E33, and Coxsackieviruses, CVA-13, CVA-20, CVA-24, were found at all six sites year-round (Fig 4). Types of Echoviruses detected in this study in Guatemala resembled similar types found in wastewater ES in a 2016 study in Haiti [14].

## Discussion

Following established poliovirus ES methods including sewage concentration, virus isolation, and the introduction of an additional cell line (i.e., HLF), a more comprehensive understanding of EV presence was achieved without a significant increase in effort or cost. In prospective testing, 61% (63/104) of sewage samples showed CPE positive results in both RD and HLF cells, indicating the presence of both PV and NPEVs. The isolates underwent whole-genome sequencing using next-generation methods on the Illumina MiSeq platform. Phylogenetic analysis of the genomic data revealed the presence of at least one EV in the sewage samples that exhibited CPE during isolation. For these EVs, either partial or complete capsid genes were resolved with genome lengths between 7,132 to 7,395 nucleotides (and near-complete genomes 7,015–7,143 nucleotides). The successful isolation and genomic typing of a diverse range of EVs, including echoviruses 1, 3, 6, 7, 11, 13, 18, 19, 25, 29; CVA-13, B2, and B5; EV-C99, EVB; and PVs (Sabin 1 and 3), from both RD and HLF cell lines confirms the effectiveness of incorporating the additional cell line into the testing algorithm [40]. The HLF cell line demonstrated its specificity by exclusively capturing echovirus 13 (E13), Coxsackievirus A13 (CVA-13), and enterovirus C99 (EV-C99) that were not found in other cell lines tested.

Environmental surveillance is conducted in countries, particularly those at high risk of circulating vaccine-derived Poliovirus (cVDPV) outbreaks [41]. Accurate identification of EVs in wastewater is dependent on the reliability and limitations of the methods employed [8]. Monitoring NPEVs in wastewater can help offer valuable data, serve as an indicator of viral circulation, and provide an early warning system for emerging viral threats. Incorporating additional cell lines, such as HLF cells alongside the RD cell line, is a possibility to enhance the detection of NPEVs, providing a more comprehensive assessment of viral diversity in wastewater. While the RD cell line is highly sensitive and widely used for enterovirus detection, it may not capture every NPEV effectively. Having additional cell lines, such as HLF cells, can potentially enhance the detection of certain NPEVs, thus providing a more comprehensive assessment of viral diversity in wastewater. Ultimately, the value of adding another cell line should be assessed considering its ability to improve the quality and reliability of NPEV surveillance data. Adding NGS can help identify recombinant EV types that have not been previously sequenced or characterized which may have public health interest. While EV ES has been performed in various countries, this study investigates the presence of EVs in a low-middle income country, which is often characterized by open sewage systems and the lack of sewage treatment plants [14,42]. Through the selection of advantageous cell lines, multiple EV types (from different genera) present in the same sewage could be resolved. Thus, enhancing ES detection systems can potentially prevent or minimize the impact of future outbreak scenarios. However, it is important to emphasize that surveillance must persist for several years even after polio elimination has been achieved [43].

This study encountered certain limitations, such as the unavailability of specific cell lines (such as MRC-5 human fetal lung fibroblast) at the CDC-cell facility and logistical challenges associated with maintaining cells at two different temperatures. Therefore, it is important to consider alternative cell lines that can be maintained at the same temperature as RD and L20B cells (i.e., 36°C). Another limitation was the small number of sample sites tested in the preliminary work. Each cell line was tested once against each site. The aim was to keep the RD cell line, and use HLF cells to supplement the existing gold standard cells. A larger sample set or a larger number of flasks tested could have provided more data to support the HLF cells as an addition or to rule it out completely. One drawback is the time and resources required for growing viruses in cells versus applying the existing highly developed direct detection methods from sewage, which were primarily developed during the COVID-19 pandemic [42]. This highlights the need for efficient and streamlined approaches to enhance the process of virus growth and detection in future studies.

In future research, it is essential to assess the reliability and effectiveness of the HLF cell line. One potential next step could involve parallel testing of all incoming environmental samples using the existing isolation algorithm for PVs. In this approach, samples displaying CPE could be extracted, and viral RNA could be sequenced to determine the presence of EVs. To enhance the sequencing effort, the utilization of Oxford Nanopore sequencing could prove beneficial. This approach has recently been employed for the detection of PV2 during the New York response in 2022, showcasing its potential in characterizing viruses quickly [44].

## Conclusion

As the Global Polio Eradication Initiative (GPEI) approaches its goal of eradicating PV, it is important to also maintain surveillance of NPEVs, which also cause AFP and other morbidities [9]. ES plays a critical role in the GPEI endgame strategy as it enables the detection of vaccine strains, wildtype PVs, and VDPVs, thereby serving as a valuable complement to AFP surveillance [14]. By incorporating an additional cell line and leveraging NGS, this study enhanced the existing poliovirus isolation algorithm, providing insights into the circulation of EVs in Guatemala without incurring extra collection or concentration costs.

This study also identified and described 21 genomic EV sequences in a low- and middle-income setting, which exhibited close genetic relatedness to sequences previously reported in Haiti [14]. The findings from this study not only contributes to our understanding of EV circulation but also enhances the monitoring of circulating EVs in the post-eradication phase when polio has been eliminated and ES activities continue.

## Supporting information

S1 Fig Microscopic appearance of the destruction of the cell monolayer. *Optical microscope images depict HLF cell monolayers at a magnification of 40×. A: Represents a monolayer with no visible cell destruction, graded as 0–1. B: Illustrates a grade 2 appearance indicating up to 50% destruction of the monolayer (cell rounding and detachment). C: Depicts a grade 3 appearance with up to 75% destruction (cell rounding and detachment). D: Shows a grade 4 appearance indicating complete destruction of the monolayer (100%). A cytopathic effect (CPE) grade of 3 or higher, representing 75% or more destruction of the monolayer, is considered indicative of the presence of the virus.
(DOCX)
