# Supplementary material for: Molecular epidemiology of enteroviruses from Guatemalan wastewater isolated from human lung fibroblasts
Source: PLoS One. 2024 Jul 3;19(7):e0305108. doi: 10.1371/journal.pone.0305108 (PMC11221682; doi:10.1371/journal.pone.0305108)
Supplement: S1 Fig — *Optical microscope images depict HLF cell monolayers at a magnification of 40×. A: Represents a monolayer with no visible cell destruction, graded as 0–1. B: Illustrates a grade 2 appearance indicating up to 50% destruction of the monolayer (cell rounding and detachment). C: Depicts a grade 3 appearance with up to 75% destruction (cell rounding and detachment). D: Shows a grade 4 appearance indicating complete destruction of the monolayer (100%). A cytopathic effect (CPE) grade of 3 or higher, representing 75% or more destruction of the monolayer, is considered indicative of the presence of the virus. (DOCX) [file pone.0305108.s001.docx]

**Supporting Information**

**S1 Figure: Microscopic appearance of the destruction of the Cell monolayer.**

Optical microscope images depict HLF cell monolayers at a magnification of 40×. A: Represents a monolayer with no visible cell destruction, graded as 0-1. B: Illustrates a grade 2 appearance indicating up to 50% destruction of the monolayer (cell rounding and detachment). C: Depicts a grade 3 appearance with up to 75% destruction (cell rounding and detachment). D: Shows a grade 4 appearance indicating complete destruction of the monolayer (100%). A cytopathic effect (CPE) grade of 3 or higher, representing 75% or more destruction of the monolayer, is considered indicative of the presence of the virus.

| 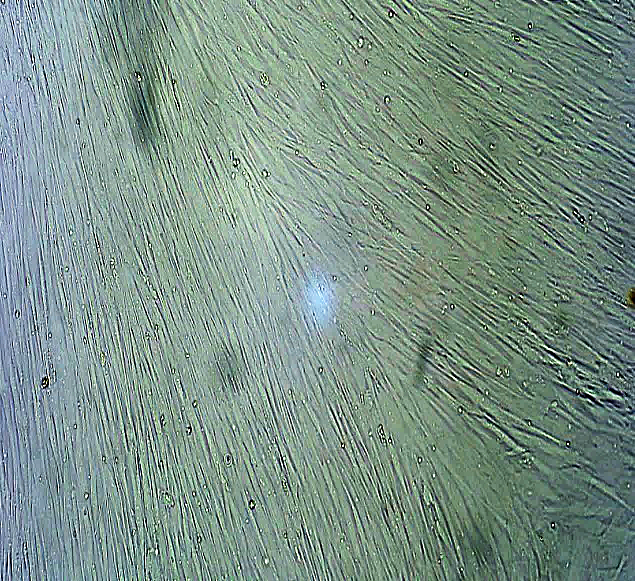  A | 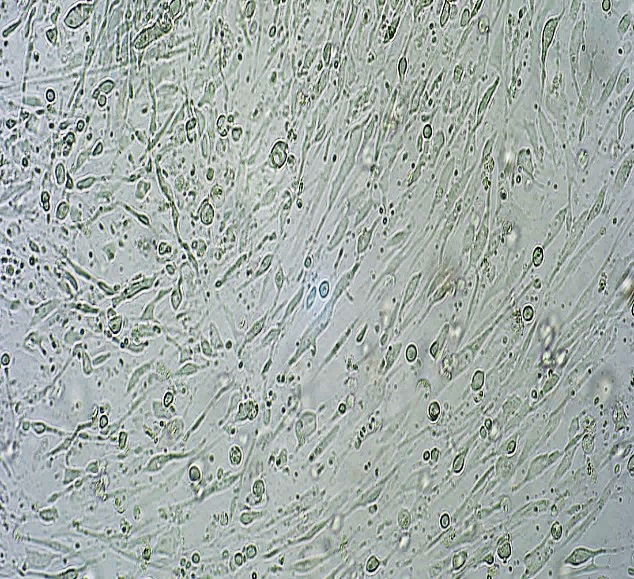  B |
| --- | --- |
| 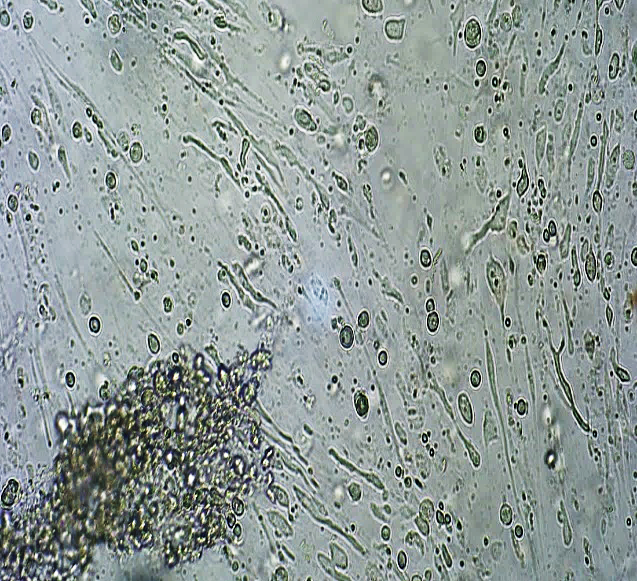  C | 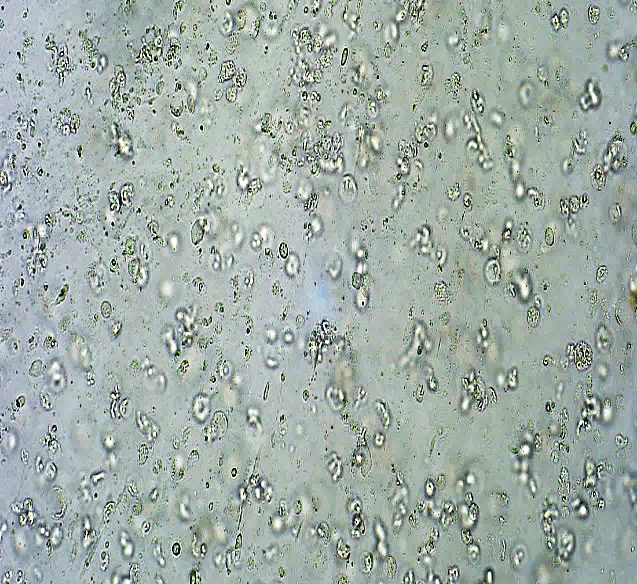  D |
